# Understanding HIV risks among adolescent girls and young women in informal settlements of Nairobi, Kenya: Lessons for DREAMS

**DOI:** 10.1371/journal.pone.0197479

**Published:** 2018-05-31

**Authors:** Abdhalah Ziraba, Benedict Orindi, Sheru Muuo, Sian Floyd, Isolde J. Birdthistle, Joyce Mumah, Jane Osindo, Pauline Njoroge, Caroline W. Kabiru

**Affiliations:** 1 African Population and Health Research Center, Manga Close, Nairobi, Kenya; 2 Katholieke Universiteit Leuven, Kapucijnenvoer 35, Leuven, Belgium; 3 Faculty of Epidemiology and Population Health, London School of Hygiene and Tropical Medicine, London, United Kingdom; 4 School of Public Health, University of Witwatersrand, Parktown, South Africa; 5 Population Council, Nairobi, Kenya; UNAIDS, UNITED STATES

## Abstract

**Introduction:**

High incidence of HIV infection among adolescent girls and young women (AGYW) has been attributed to the numerous and often layered vulnerabilities that they encounter including violence against women, unfavourable power relations that are worsened by age-disparate sexual relations, and limited access to sexual and reproductive health information and services. For AGYW living in urban informal settlements (slums), these vulnerabilities are compounded by pervasive poverty, fragmented social networks, and limited access to social services including health and education. In this paper, we assess sexual risk behaviours and their correlates among AGYW in two slum settlements in Nairobi, Kenya, prior to the implementation of interventions under the Determined Resilient Empowered AIDS-free Mentored and Safe (DREAMS) Partnership.

**Methods:**

We drew on secondary data from the Transition to Adulthood study, the most recent representative study on adolescent sexual behaviour in the two settlements. The study was nested within the Nairobi Urban Health and Demographic Surveillance System (NUHDSS). Data were collected in 2009 from 1,390 AGYW aged 12–23 years. We estimated the proportions of AGYW reporting ever tested for HIV, condom use, multiple sexual partners and age-disparate sex by socio-demographic characteristics. “High risk” sexual behaviour was defined as a composite of these four variables and age at first sex. Multivariable regression analyses were performed to identify factors associated with risk behaviours.

**Results:**

Fifty-one percent of AGYW reported that they had ever tested for HIV and received results of their last test, with the proportion rising steeply by age (from 15% to 84% among those <15 years and 20–23 years, respectively). Of 578 AGYW who were sexually active in the 12 months preceding the survey, 26% reported using a condom at last sex, 4% had more than one sexual partner, and 26% had sex with men who were at least 5 years older or younger. All girls aged below 15 years who had sex (n = 9) had not used condoms at last sex. The likelihood of engaging in “high risk” sexual risk behaviour was higher among older AGYW (19–23 years), those in marital unions, of Luo ethnicity, out of school, living alone or with a friend (versus parents), living with spouse (versus parents), and those whose friends engaged in risky/anti-social behaviours. In contrast, Muslim faith, co-residence with both parents, and belonging to an organised social group were associated with lower odds of risky sexual behaviours.

**Conclusion:**

Our study findings suggest that multifaceted approaches addressing the educational and social mediators of AGYW’s vulnerability and that also reach the people with whom AGYW live and interact, are needed to reduce the rapid onset of sexual risk during the adolescent years. There is a particular need to reach the youngest adolescent girls in poor urban settings, among whom condom use and awareness of HIV status is rare.

## Introduction

Globally, adolescent girls and young women (AGYW) aged 15 to 24 years accounted for 20% of new HIV infections among people aged 15 years and older in 2015 [1]. In sub-Saharan Africa (SSA), the proportion was higher, with 25% of new HIV infections occurring among AGYW [1]. Kenya is among the top four countries hit hardest by the epidemic with 1.5 million people living with HIV/AIDS and nearly 36,000 deaths due to AIDS related illnesses in the year 2015 [2]. Residents of Nairobi’s informal settlements (slums) are among the most affected by HIV in Kenya. HIV prevalence is higher among slum residents compared with those living in non-slum parts of Nairobi city [3]. A serological survey conducted in two slums of Nairobi reported an HIV prevalence of 12%, which was twice the prevalence observed in rural (6%) and also higher than levels in urban areas (5%) of Kenya [3].

HIV transmission among AGYW in SSA is mainly through the heterosexual route [4,5]. AGYW in Kenya are two times more likely to be HIV positive than their male counterparts [6]. Economic hardship in the slums is thought to increase women’s involvement in sex for financial reasons and to access basic needs like food and clothing for themselves and their families [7]. The odds of HIV infection for women who reported that they have had sex for money, gifts or favours are five times higher than those who do not report having sex for these reasons [8]. This is partly because they have male sexual partners who are often much older, more sexually experienced and have a higher risk profile [7]. Often, despite being aware of the risks, AGYW are not able to negotiate for safer sex such as condom use due to unfavourable power relations [9,10], which increase their risk of HIV infection [11,12]. A cross-sectional study by Hunter et al. reported an increased risk of HIV among young women with multiple sex partners [13].

High risk sex remains a key factor in HIV transmission. Distally, there are important factors that predispose AGYW to high risk sex including lack of prevention and treatment information, limited access to services and societal norms that predispose girls and women to violence and early marriages. High risk sex also increases the risk of other sexually transmitted infections (STIs) that in turn increase the risk of HIV acquisition [14]. Early sexual debut among women in the slums has been reported, with median age at first sex as low as 15 years [7,15]. In a study in Kenya in 2012, having first sex after the age of 19 years was associated with a 62% lower odds of being HIV positive compared to women who first had sex before 15 years [3].

HIV counselling and testing is crucial in the prevention and treatment of HIV. A household survey conducted in two urban slums of Nairobi showed that AGYW were less aware of their HIV status compared to women between the ages of 25 and 34 years [16]. Studies have shown a strong association between HIV risk perception and awareness of partners’ HIV status[8]. Women who experience partner violence may be at higher risk for HIV infection. A study in Nairobi found that HIV positive women were almost twice as likely to experience physical violence compared to HIV negative women [17].

With respect to Nairobi city slums, there are significant data gaps on HIV risk among AGYW in the context of poverty, trends in new infections and uptake of prevention and treatment services. Nevertheless, major public health interventions to decrease HIV acquisition among AGYW have been rolled out, including the Determined Resilient Empowered AIDS-free Mentored and Safe (DREAMS) Partnership [18]. In this paper, we describe the sexual behaviour profiles of AGYW as well as the factors associated with high risk sexual behaviour using data from the Transition to Adulthood Survey the most recent representative survey conducted pre-DREAMS within the Nairobi Urban Health and Demographic Surveillance System (NUHDSS). Estimates from this study will provide a picture of sexual risk behaviours and associated factors among AGYW living in Nairobi’s informal settlements prior to DREAMS roll-out, and a reference with which to track change during DREAMS implementation over time.

## Methods

### Study design, setting and sample

The Transition to Adulthood (TTA) study [19] was nested within the NUHDSS, a longitudinal platform managed by the African Population and Health Research Center (APHRC) since 2002. The NUHDSS covers Korogocho and Viwandani informal settlements, located about 7 kilometers from each other and covering a total area of about 0.97 km^2^. The two slums are characterized by high levels of unemployment, sub-standard housing and crowding, limited access to education and other social services, high levels of insecurity, and inadequate water and sanitation infrastructure. However, the two slums have markedly different demographic, economic, and health indicators. Korogocho is a more settled community with many long-term residents while the population in Viwandani is more mobile and youthful [20].

The TTA study design and data collection procedures are described elsewhere [19]. In brief, the study aimed to identify protective and risk factors among a representative sample of young people in the NUHDSS aged 12–24 years; and how these factors influenced young people’s transition to adulthood.

From November 2007 through June 2008, young people aged 12–24 years were interviewed as part of Wave 1 of TTA. In 2009 and 2010, respondents were re-interviewed in two additional waves (i.e., Wave 2 and Wave 3). Data are available for 3,981 individuals (Wave1), 2,659 (Wave 2) and 1,910 (Wave 3). During the second and third waves of data collection, attempts were also made to include adolescents who were not traced in the earlier waves, and additional questions were included. The present analysis focuses on Wave 2 as it has interview questions of interest to the evaluation of DREAMS; we restricted our analysis to female respondents aged 12 to 23 years (given DREAMS prioritisation of AGYW aged 10–24 years). All these data and relevant information can be accessed at the APHRC Microdata Portal [19] and are included with this manuscript within the supplementary files (S7 Table).

### Measures

Explanatory variables: Explanatory variables included slum of residence (i.e., Korogocho = 1, Viwandani = 2), age of respondent at survey (12–14 years = 1, 15–19 years = 2, 20–23 years = 3), marital status as at the survey (1 if married, and 0, otherwise), religion (1 if Catholic, 2 if Protestant, 3 if Pentecostal, 4 if other Christian, 5 if Muslim, and 6 if no religion), schooling (0 if currently in school, 1 if never attended school or incomplete primary, 2 if completed primary, 3 if incomplete secondary, 4 if completed secondary, 5 if attained tertiary education level); ethnicity (Kikuyu, Luhya, Luo, Kamba, Kisii, Garre, and other), and wealth status, a composite measure derived using principal components analysis (PCA) with input binary variables on ownership/possession of household items such as TV, radio, bicycle motorcycle, and nature of their housing. Wealth status was grouped into three categories: lowest, middle and highest.

Mediating Variables: Participation in social group or club (whether the person belongs to any organised social group or club, e.g., religious group, drama group, anti-AIDS group, anti-drugs groups, girl guides/scout, wildlife society, self-help group, etc.), peer influence, relationship with parents/guardians, and whether the AGYW does any unpaid voluntary work in the community (e.g., cleaning the neighbourhood). Perceived involvement of peers in risk (anti-social) behaviour was measured using six items (Box 1). Responses were reported on a 4-point Likert scale (none of them = 1, some of them = 2, most of them = 3, all of them = 4). Relationship with parents/guardians was measured using four items (Box 1) on a 4-point Likert scale (“never”, “sometimes”, “most of the time”, “all the time”). For peer involvement in risk behaviour, we contrasted those who reported “none of them” and those who reported “some of them”, “most of them”, or “all of them”. For close relationship with parent, we contrasted those who reported “all of the time” or “most of the time” to those who reported “sometimes” and “never”.

**Box 1. Exact wording of items related to peer involvement in risk behaviour and relationship with parents/guardians.**

**Table:** 

**How many of your friends do/did the following?**
Drink alcohol
Run away from home
Get into trouble with the police
Have sexual intercourse
Get/Got into trouble at school (e.g. disciplinary action, get into fights, etc.)
Use drugs like bhang, khat, glue
**Relationship with your parents/guardians and how you get along**
How often do your parents/guardians encourage you to do what you are interested in doing and show an interest in those things themselves?
How often are your parents interested in what you think and feel?
How often do your parents try to find activities that you would enjoy doing after school or weekends?
When you have problems, how often can you talk them over with your parents?

Outcome Variables: The outcomes of interest were ever tested for HIV (1 if ever tested for HIV and received results of their last test, and, 0, otherwise); condom use during last sexual intercourse (1 if yes, 0 if no); recent multiple sex partners (0 if had only one sexual partner in the last 12 months, 1 if had two or more sexual partners in the past 12 months); age-disparate relationships (1 if the male partner was 0 to 4 years younger or older, 2 if the age difference was 5 to 9 years, and, 3 if the age difference was 10 years or more); and age at sexual debut.

Further, we classified high risk sexual behaviour as a composite of the above five measures. For this composite, we transformed the components as follows, to assign a higher score to responses that denote higher risk. For condom use, the responses were coded 0 if the participants never had sex, 1 if used condoms during the last sexual intercourse, and 2 if did not use condoms during the last sexual intercourse. Regarding number of sex partners, the responses were coded 0 if the participants never had sex, 1 if the number reported was 1, and 2 if the number reported was 2 or more. Age disparity at last sex was coded 0 if the person never had sex, 1 if the male partner was 0 to 4 years younger or older, 2 if the age difference was 5 to 9 years, and, 3 if the age difference was 10 years or more. Age at sexual debut was coded 0 if the person never had sex, 1 if aged 18 or older, 2 if aged 15 to 17 years, and 3 if aged 14 or younger. Ever tested for HIV was coded 0 if the person had ever been tested for HIV and received their result, and 1 otherwise. The total score of these five measures was then used as a measure of sexual risk behaviour, with higher scores indicating higher risk sexual behaviour (Cronbach’s alpha for internal consistency = 0.92).

### Statistical analysis

The analyses were performed using STATA v14 (StataCorp, College Station, TX). Descriptive analyses were conducted to estimate the proportions of AGYW reporting the following outcomes: ever tested for HIV, condom use, multiple sexual partnerships, and age disparate sex. For age at sexual debut, we estimated the median and the associated interquartile range (IQR). We summarized these variables by the socio-demographic characteristics of AGYW (age, education, ethnicity, marital status, and religion and wealth status) and slum of residence. Regression analyses were performed to assess the association between the explanatory variables and each outcome. We adopted a step-by-step construction of the model, adjusting first for socio-demographic factors and then for mediating factors. First, to screen potential risk factors for each outcome, a simple univariable association was tested for each explanatory variable with the outcome of interest (Model 1). In all subsequent models, age and slum area were considered *a priori* confounding variables and were therefore included. Socio-demographic variables whose age- and slum-adjusted associations (Model 2) were statistically significant at *p*<0.10 were included into a multivariable regression model; and those remaining statistically significant at *p*<0.10 were retained in a “core” model (Model 3). Next, the mediating variables were added to this core model one at a time. Those that were statistically significant at *p*<0.10 after adjusting for age, site and socio-demographic variables were included in a multivariable model and were retained if they remained significant at *p*<0.10 after adjustment for other mediating variables. Depending on the nature of the outcome, different model families were considered. “Ever tested for HIV” and “condom use” were analysed using logistic regression models, while an ordered logistic regression model was used for “age disparate sex”. For the high risk behaviour composite, a linear regression model was fitted. We note that i) analyses for age disparate sex, condom use, multiple sexual partners, and age at sexual debut were restricted to individuals who reported that they had ever had sex; and ii) as the number of AGYW in the age group 12–14 who reported having sex were very few (n = 9), they were not included in the models for these outcomes.

### Ethical considerations

Ethical clearance for the TTA study was obtained from the Kenya Medical Research Institute’s ethical review board. Signed or verbal consent was obtained from all respondents. For respondents aged 12–17 years, parental consent was also obtained.

## Results

### Socio-demographic characteristics of the AGYW in Nairobi’s informal settlements

The median age of AGYW was 18 years (IQR: 15–20). Table 1 presents the distribution of respondents by demographic characteristics categorised by age. More than half (54.2%) of the AGYW were from Viwandani. Most of the respondents (81.8%) were unmarried at the time of the survey, with the proportion of married AGYW increasing by age. The majority of AGYW (82.4%) were Christians, and those with no religious affiliation comprised 4.8%. Almost all girls (98.9%) in the 12–14 age bracket and 14.8% of those aged 20–23 were still in school. Overall, only 3.2% had completed tertiary education level. Kikuyu (35.8%), Kamba (17.1%) and Luo (14.9%) were the biggest ethnic groups (S1 and S2 Tables). Forty-five percent of AGYW lived with both parents; however, the proportion of AGYW living with both parents varied significantly across the age groups. Among those aged 20–23 years, 42.5% reported that they were living with spouses. (S1 and S2 Tables)

**Table 1:** Socio-demographic characteristics of the AGYW in Nairobi’s informal settlements, by age.

	All AGYW	12–14 years	15–19 years	20–23 years
	N = 1390	n = 267	n = 650	n = 473
**Slum area**				
Korogocho	637 (45.8)	105 (39.3)	339 (52.2)	193 (40.8)
Viwandani	753 (54.2)	162 (60.7)	311 (47.9)	280 (59.2)
**Marital Status**				
Unmarried	1137 (81.8)	266 (99.6)	604 (92.9)	267 (56.5)
Currently married	253 (18.2)	1 (0.4)	46 (7.1)	206 (43.6)
**Religion**				
Catholic	394 (28.4)	82 (30.7)	184 (28.3)	128 (27.1)
Protestant	265 (19.1)	49 (18.4)	119 (18.3)	97 (20.5)
Pentecostal	315 (22.7)	59 (22.1)	150 (23.1)	106 (22.4)
Other Christian	169 (12.2)	33 (12.4)	74 (11.4)	62 (13.1)
Muslim	181 (13.0)	35 (13.1)	95 (14.6)	51 (10.8)
No Religion	66 (4.8)	9 (3.4)	28 (4.3)	29 (6.1)
**Schooling**				
Currently in school	794 (57.1)	264 (98.9)	460 (70.8)	70 (14.8)
None/incomplete primary	174 (12.5)	0 (0.0)	59 (9.1)	115 (24.3)
Complete primary	175 (12.6)	1 (0.4)	57 (8.8)	117 (24.7)
Incomplete secondary	120 (8.6)	2 (0.8)	49 (7.5)	69 (14.6)
Complete secondary	68 (4.9)	0 (0.0)	12 (1.9)	56 (11.8)
Tertiary	44 (3.2)	0 (0.0)	7 (1.1)	37 (7.8)
Missing	15 (1.1)	0 (0.0)	6 (0.9)	9 (1.9)
**Ethnicity**				
Kikuyu	498 (35.8)	95 (35.6)	239 (36.8)	164 (34.7)
Luhya	159 (11.4)	22 (8.2)	84 (12.9)	53 (11.2)
Luo	207 (14.9)	45 (16.9)	105 (16.2)	57 (12.1)
Kamba	238 (17.1)	43 (16.1)	88 (13.5)	107 (22.6)
Kisii	71 (5.1)	16 (6.0)	22 (3.4)	33 (7.0)
Garre	70 (5.0)	9 (3.4)	41 (6.3)	20 (4.2)
Other	147 (10.6)	37 (13.9)	71 (10.9)	39 (8.3)
**Wealth tertile**				
Lowest	304 (21.9)	49 (18.4)	148 (22.8)	107 (22.6)
Middle	387 (27.8)	68 (25.5)	178 (27.4)	141 (29.8)
Highest	642 (46.2)	144 (53.9)	298 (45.9)	200 (42.3)
Missing	57 (4.1)	6 (2.3)	26 (4.0)	25 (5.3)
**Living arrangements**				
Live with 1 parent	344 (24.8)	61 (22.9)	199 (30.6)	84 (17.8)
Both parents	618 (44.5)	192 (71.9)	333 (51.2)	93 (19.7)
Guardian	90 (6.5)	13 (4.9)	55 (8.5)	22 (4.7)
Alone or with friend	58 (4.2)	0 (0.0)	11 (1.7)	47 (9.9)
Spouse	245 (17.6)	1 (0.4)	43 (6.6)	201 (42.5)
Other	35 (2.5)	0 (0.0)	9 (1.4)	26 (5.5)

### High risk sexual behaviour outcomes

Table 2 shows the proportions of AGYW who reported various sexual behaviours by age category. Overall, 41.6% had ever had sex, and by age group, the highest proportion was among the oldest group: 20–23 year olds (80.1%). Just over half of AGYW (52%) reported that they had ever been tested for HIV and received results of their last test, with the proportion varying from 14.6% among those younger than 15 years to 82% among those aged 20–23 years. Of the 578 AGYW who were sexually active in the 12 months preceding the survey, 26% indicated that they used condoms at last sex with their most recent partner. This proportion was greater (30.5%) among those aged 15–19 years, than those aged 20–23 years (23.8%) and 12–14 years (0.0%). For the majority (55.7%) of those who had sex, the age difference between them and the last sexual partner was less than 5 years and another 35.5% had sexual partners with an age difference of 5 to 9 years. About one in twenty of the 578 AGYW who had ever had sex reported that they had more than one sexual partner in the 12 months preceding the survey. The median age at first sexual encounter, among those who had ever had sex, was 16 years (IQR: 15 to 18).

**Table 2:** Distribution of outcome variables for risky sexual behaviour for AGYW by age.

Outcome variables	All AGYW	12–14 years	15–19 years	20–23 years
	N = 1390	n = 267	n = 650	n = 473
**Ever had sex**				
No	807 (58.1)	257 (96.3)	458 (70.5)	92 (19.5)
Yes	578 (41.6)	9 (3.4)	190 (29.2)	379 (80.1)
Missing	5 (0.4)	1 (0.4)	2 (0.3)	2 (0.4)
**Ever tested for HIV and received results for that test**				
Never tested	668 (48.1)	228 (85.4)	357 (54.9)	83 (17.6)
Ever tested and received result	718 (51.7)	39 (14.6)	292 (44.9)	387 (81.8)
Refused	4 (0.3)	0 (0.0)	1 (0.2)	3 (0.6)
	All females	12–14 years	15–19 years	20–23 years
	N = 578	n = 9	n = 190	n = 379
Age disparity of last sexual partner*				
0–4	322 (55.7)	5 (55.6)	102 (53.7)	215 (56.7)
5–9	205 (35.5)	2 (22.2)	63 (33.2)	140 (36.9)
10–15	29 (5.0)	0 (0.0)	13 (6.8)	16 (4.2)
Missing	22 (3.8)	2 (22.2)	12 (6.3)	8 (2.1)
Condom use at last sex*				
Yes	148 (25.6)	0 (0.0)	58 (30.5)	90 (23.8)
No	408 (70.6)	7 (77.8)	122 (64.2)	279 (73.6)
Missing	22 (3.8)	2 (22.2)	10 (5.3)	10 (2.6)
Number of sex partners in the past 12 months (i.e. n = 481)*				
1	458 (95.2)	5 (100.0)	133 (95.0)	320 (95.2)
2 to 7	23 (4.8)	0 (0.0)	7 (5.0)	16 (4.8)
Age at sex debut*				
Median (IQR) years	16 (15–18)	12.5 (11–13)	15 (14–17)	17 (16–19)

*Restricted to those who have ever had sex

### Mediators for high risk sexual behaviour

Table 3 shows the distribution of social mediating variables, including belonging to a social group, peer involvement in risky behaviours and relationship with parents or guardians. Overall, 56.0% of AGYW belonged to a social group. The proportions were higher in the 12–14 years age group (75.3%) compared to 60.0% and 39.5% among the 15–19 and 20-23-year-old AGYW, respectively. While a large proportion (44.1%) of the AGYW reported that all or most of their friends were involved in at least two of the six risk behaviours, the proportion was low among 12-14-year-olds and increased with age. Among those aged 12–14 years, about 51% indicated their friends were involved in only one of the six behaviours. Relationships with parents or guardians were closest among 12-14-year-olds, among whom about 82% had a positive response to two or more of the four questions.

**Table 3:** Social mediators of high risk behaviour, by age.

Mediating variables	All AGYW	12-14years	15-19years	20-23years
	N = 1390	N = 267	N = 650	N = 473
Belongs to any group**				
No	612 (44.0)	66 (24.7)	260 (40.0)	286 (60.5)
Yes	778 (56.0)	201 (75.3)	390 (60.0)	187 (39.5)
Peer involvement in risk behaviour*				
Yes to none	292 (21.0)	82 (30.7)	134 (20.6)	76 (16.1)
Yes to 1 item	485 (34.9)	136 (50.9)	235 (36.2)	114 (24.1)
Yes to 2 or more items	613 (44.1)	49 (18.4)	281 (43.2)	283 (59.8)
**Close relationship with parents/guardians**				
Yes no none	445 (32.0)	22 (8.2)	121 (18.6)	302 (63.9)
Yes to 1 item	148 (10.7)	24 (9.0)	87 (13.4)	37 (7.8)
Yes to 2 or more items	797 (57.3)	221 (82.8)	442 (68.0)	134 (28.3)
**Does voluntary work in the community**				
No	747 (53.7)	134 (50.2)	334 (51.4)	279 (59.0)
Yes	643 (46.3)	133 (49.8)	316 (48.6)	194 (41.0)

**Groups comprise religious group, drama group, Anti-AIDS, Anti-drugs, girl guides/scout, wild life society, self-help, etc

*Contrasting some of them, most of them, and all of them to none of them.

### Risk factors for high risk sexual behaviour among AGYW

Table 4 shows results from our final logistic regression model assessing the association between HIV testing and the socio-demographic characteristics. Generally, older AGYW (15–23 years) were more likely to have ever tested for HIV than younger AGYW (12–14 years). Compared to 12-14-year-old AGYW, those aged 15–19 years had about three times higher odds of testing and this was even higher for the 20-23-year-olds (AOR = 7.1, 95%CI: 4.28–11.69). Schooling status was associated with the likelihood of testing. Those not currently in school and had no/incomplete primary, completed primary, and incomplete secondary education were, respectively, twice, three times and two and half times more likely to have tested for HIV than those currently in school (None/incomplete primary: AOR = 1.86, 95%CI: 1.12–3.09; Complete primary: AOR = 2.65, 95%CI: 1.63–4.30; Incomplete secondary: AOR = 2.36, 95%CI: 1.44–3.88). Compared to those living with one parent, AGYW living with both parents were less likely to have ever tested (AOR = 0.55; 95%CI: 0.41–0.75), while those living with spouses were about three times more likely to test for HIV (AOR = 3.18, 95%CI: 1.78–5.70). AGYW whose friends were involved in two or more of the six risk behaviours were more likely to have ever tested for HIV (AOR = 2.01, 95%CI: 1.40–2.88). Being involved in voluntary work in the community was associated with significantly higher odds of having ever tested for HIV. Results from all four regression models (unadjusted, and with three sets of adjustments) are presented as supplementary information (S3 through S6 Tables).

**Table 4:** Factors associated with HIV testing among AGYW aged 12–23 years.

	Ever tested for HIV			
	Number who ever tested for HIV / N (%)	Model2 AOR (95%CI)	Model4 AOR (95%CI)	Pvalue
**Age (years)**		*p*<0.001	*p*<0.0001	
12–14	39/267 (14.6)	1	1	
15–19	292/650 (44.9)	4.74 (3.26–6.89)	2.89 (1.95–4.29)	<0.001
20–23	387/473 (81.8)	27.25 (18.01–41.24)	7.08 (4.28–11.69)	<0.001
**Slum area**		*p* = 0.530	*p* = 0.523	
Korogocho	331/637 (52.0)	1	1	
Viwandani	387/753 (51.4)	0.92 (0.72–1.18)	0.92 (0.70–1.20)	0.523
**Marital Status**		*p* <0.001		
Unmarried	490/1137 (43.1)	1		
Currently married	228/253 (90.1)	4.83 (3.05–7.65)		
**Religion**		*p* = 0.005		
Catholic	203/394 (51.5)	1		
Protestant	141/265 (53.2)	0.98 (0.68–1.40)		
Pentecostal	171/315 (54.3)	1.12 (0.80–1.58)		
Other Christian	96/169 (56.8)	1.14 (0.75–1.75)		
Muslim	69/181 (38.1)	0.49 (0.32–0.75)		
No Religion	38/66 (57.6)	0.94 (0.51–1.72)		
**Schooling**		*p*<0.001	*p*<0.001	
Currently in school	252/794 (31.7)	1	1	
None/incomplete primary	140/174 (80.5)	3.59 (2.31–5.58)	1.86 (1.12–3.09)	0.017
Complete primary	142/175 (81.1)	4.00 (2.54–6.30)	2.65 (1.63–4.30)	<0.001
Incomplete secondary	89/120 (74.2)	2.93 (1.82–4.71)	2.36 (1.44–3.88)	0.001
Complete secondary	52/68 (76.5)	2.26 (1.19–4.27)	1.93 (0.99–3.75)	0.055
Tertiary	34/44 (77.3)	2.21 (1.02–4.8)	2.04 (0.92–4.53)	0.080
**Ethnicity**		*p* = 0.005		
Kikuyu	271/496 (54.6)	1		
Luhya	85/158 (53.8)	0.90 (0.6–1.35)		
Luo	110/209 (52.6)	1.00 (0.69–1.46)		
Kamba	132/239 (55.2)	0.84 (0.58–1.23)		
Kisii	35/70 (50.0)	0.69 (0.37–1.29)		
Garre	24/70 (34.3)	0.33 (0.18–0.60)		
Other	61/148 (41.2)	0.6 (0.39–0.92)		
**Wealth status**		*p* = 0.052		
Lowest	181/311 (58.2)	1		
Middle	194/373 (52.0)	0.70 (0.50–1.00)		
Highest	309/651 (47.5)	0.69 (0.5–0.94)		
**Living arrangements**		*p*<0.001	*p*<0.001	
One parent	181/344 (52.6)	1	1	
Both parents	202/618 (32.7)	0.51 (0.38–0.69)	0.55 (0.41–0.75)	<0.001
Guardian	42/90 (46.7)	0.73 (0.44–1.19)	0.74 (0.44–1.23)	0.244
Alone or with friend	42/58 (72.4)	1.15 (0.58–2.28)	0.83 (0.41–1.68)	0.600
Spouse	221/245 (90.2)	3.87 (2.31–6.47)	3.18 (1.78–5.70)	<0.001
Other	30/35 (85.7)	2.59 (0.95–7.06)	1.72 (0.61–4.81)	0.304
**Belongs to any group?**		*p* = 0.038		
No	377/612 (61.6)	1		
Yes	341/778 (43.8)	0.77 (0.60–0.99)		
**Peer influence**		*p*<0.001	*p*<0.001	
Yes no none	112/292 (38.4)	1	1	
Yes to 1 item	197/485 (40.6)	1.19 (0.85–1.68)	1.17 (0.82–1.68)	0.393
Yes to 2 or more items	409/613 (66.7)	2.32 (1.66–3.25)	2.01 (1.4–2.88)	<0.001
**Relationship with parents/guardians**		*p*<0.001		
Yes no none	337/445 (75.7)	1		
Yes to 1 item	63/148 (42.6)	0.41 (0.26–0.63)		
Yes to 2 or more items	318/797 (39.9)	0.48 (0.35–0.65)		
**Does voluntary work in the community**		*p* = 0.181	*p* = 0.014	
No	386/747 (51.7)	1	1	
Yes	332/643 (51.6)	1.18 (0.93–1.51)	1.39 (1.07–1.81)	0.014

Model 2: Age- and site-adjusted model for each covariate with *p*<0.10 in Model 1; Model 4: Age, site and socio-demographic adjusted multivariable model including mediating variables with *p*<0.1 after adjusting for Model 3 variables. OR is odds ratio; AOR is adjusted OR.

Table 5 shows the results from a logistic regression model assessing the association between the various socio-demographic characteristics and the mediating factors (belonging to a social group, peer involvement in risk behaviour, relationship with parents and involvement in voluntary work in the community) with condom use at last sex, among AGYW who reported to have ever had sex. It shows that after adjusting for age and slum of residence, being married was strongly associated with lower odds of condom use at last sex, while there was no evidence that other socio-demographic or mediating factors were associated with this outcome.

**Table 5:** Factors associated with condom use among AGYW aged 15–23 years.

Variables	Condom use			
	Used condoms / N (%)	Model 2 AOR (95%CI)	Model 4 AOR (95%CI)	Pvalue
**Age (years)**		*p* = 101	*p* = 0.430	
15–19	58/190 (30.5)	1	1	
20–23	90/379 (23.7)	0.72 (0.48–1.07)	1.19 (0.77–1.84)	0.430
**Slum area**		P = 103	*p* = 0.858	
Korogocho	79/264 (29.9)	1	1	
Viwandani	69/305 (22.6)	0.73 (0.49–1.07)	1.04 (0.68–1.59)	0.858
**Marital status**		*p*<0.001	*p*<0.001	
Unmarried	131/318 (41.2)	1	1	
Currently married	17/251 (6.8)	0.09 (0.05–0.16)	0.09 (0.05–0.16)	<0.001
**Religion**		*p* = 0.0625		
Catholic	54/172 (31.4)	1		
Protestant	31/107 (29)	0.84 (0.49–1.44)		
Pentecostal	34/139 (24.5)	0.7 (0.42–1.18)		
Other Christian	20/73 (27.4)	0.79 (0.42–1.48)		
Muslim	1/43 (2.3)	0.05 (0.01–0.36)		
No Religion	8/35 (22.9)	0.59 (0.25–1.41)		
**Schooling**		*p*<0.001		
Currently in school	48/104 (46.2)	1		
None/incomplete primary	24/155 (15.5)	0.2 (0.11–0.37)		
Complete primary	38/149 (25.5)	0.37 (0.21–0.66)		
Incomplete secondary	18/75 (24)	0.37 (0.18–0.74)		
Complete secondary	12/47 (25.5)	0.42 (0.19–0.95)		
Tertiary	7/28 (25)	0.39 (0.15–1.06)		
**Ethnicity**		*p* = 0.7361		
Kikuyu	59/211 (28)	1		
Luhya	20/75 (26.7)	1.01 (0.55–1.85)		
Luo	30/96 (31.3)	1.07 (0.63–1.82)		
Kamba	26/110 (23.6)	0.97 (0.55–1.71)		
Kisii	7/27 (25.9)	1.18 (0.45–3.07)		
Garre	0/11 (0)	1 (0–0)		
Other	6/39 (15.4)	0.49 (0.19–1.24)		
**Wealth status**		*p* = 0.8037		
Lowest	32/137 (23.4)	1		
Middle	48/165 (29.1)	1.15 (0.68–1.94)		
Highest	63/238 (26.5)	1.00 (0.61–1.64)		
**Living arrangements**		*p*<0.001		
One parent	59/126 (46.8)	1		
Both parents	42/103 (40.8)	0.80 (0.46–1.37)		
Guardian	5/23 (21.7)	0.34 (0.12–10)		
Alone or with friend	15/44 (34.1)	0.50 (0.24–1.04)		
Spouse	16/244 (6.6)	0.07 (0.03–0.12)		
Other	11/29 (37.9)	0.58 (0.25–1.34)		
**Belongs to any group?**		*p* = 0.3420		
No	92/365 (25.2)	1		
Yes	56/204 (27.5)	1.21 (0.82–1.80)		
**Peer influence**		*p* = 0.3989		
Yes no none	17/74 (23)	1		
Yes to 1 item	27/128 (21.1)	0.77 (0.38–1.56)		
Yes to 2 or more items	104/367 (28.3)	1.08 (0.59–1.98)		
**Relationship with parents/guardians**		*p*<0.001		
Yes no none	56/347 (16.1)	1		
Yes to 1 item	14/40 (35)	3.22 (1.54–6.76)		
Yes to 2 or more items	78/182 (42.9)	4.22 (2.70–6.60)		
**Does voluntary work in the community**		*p* = 0.187		
No	81/342 (23.7)	1		
Yes	67/227 (29.5)	1.30 (0.88–1.91)		

Model 2: Age- and site-adjusted model for each covariate with *p*<0.10 in Model 1; Model 4: Age, site and socio-demographic adjusted multivariable model including mediating variables with *p*<0.1 after adjusting for Model 3 variables. OR is odds ratio; AOR is adjusted OR. No mediating variable made it to the final model as such Model 4 is the same as Model 3.

Table 6 summarizes results from an ordered logistic regression model assessing the factors associated with having sex with younger or older male partners among AGYW aged between 15 and 23 years. Compared to the younger girls (15–19 years), the older AGYW (20–23 years) had about twice the odds of having had sex with males who were much older or younger. The results show that married AGYW were substantially less likely to have sex with much older or younger male partners, than their unmarried counterparts. AGYW whose friends were involved in two or more of the six risk behaviours had 60% lower odds of having sex with much older or younger men.

**Table 6:** Factors associated with age disparate sex among AGYW aged 15–23 years.

Variables	N (% whose partner's age difference was (0-4yr)(5-9yrs)(10+ yrs))	Age disparity at last sex+		
		Model2 AOR (95%CI)	Model4 AOR (95%CI)	Pvalue
**Age(years)**		P = 0.355	P = 0.005	
15–19	190 (53.7)(33.2)(6.8)	1	1	
20–23	379 (56.7)(36.9)(4.2)	1.19 (0.83–1.71)	1.78 (1.19–2.65)	0.005
**Slum area**		P = 0.001	P = 0.011	
Korogocho	264 (62.9)(28.4)(4.9)	1	1	
Viwandani	305 (49.5)(42.0)(5.2)	0.56 (0.4–0.8)	0.62 (0.43–0.9)	0.001
**Marital Status**		P<0.001	P<0.001	
Unmarried	318 (65.7)(26.4)(2.2)	1	1	
Currently married	251 (43.0)(47.4)(8.8)	0.30 (0.21–0.44)	0.28 (0.19–0.41)	<0.001
**Religion**		P = 0.3453		
Catholic	172 (60.5)(31.4)(3.5)	1		
Protestant	107 (58.9)(34.6)(4.7)	0.85 (0.51–1.4)		
Pentecostal	139 (48.9)(46.0)(2.9)	0.68 (0.43–1.07)		
Other Christian	73 (54.8)(34.2)(5.5)	0.70 (0.39–1.24)		
Muslim	43 (51.2)(25.6)(18.6)	0.48 (0.24–0.99)		
No Religion	35 (57.1)(34.3)(5.7)	0.73 (0.34–1.54)		
**Schooling**		P = 0.0025		
Currently in school	104 (64.4)(24.0)(2.9)	1		
None/incomplete primary	155 (45.2)(45.8)(5.8)	0.38 (0.22–0.68)		
Complete primary	149 (53.7)(40.9)(4.0)	0.49 (0.28–0.87)		
Incomplete secondary	75 (53.3)(40.0)(5.3)	0.56 (0.29–1.08)		
Complete secondary	47 (70.2)(21.3)(8.5)	1.03 (0.46–2.32)		
Tertiary	28 (67.9)(21.4)(0.0)	1.15 (0.41–3.27)		
**Ethnicity**		P = 0.1794		
Kikuyu	211 (58.8)(34.6)(3.3)	1		
Luhya	75 (52.0)(36.0)(4.0)	0.97 (0.56–1.69)		
Luo	96 (57.3)(37.5)(4.2)	0.83 (0.51–1.36)		
Kamba	109 (57.8)(36.7)(2.8)	1.22 (0.74–2.02)		
Kisii	27 (51.9)(40.7)(7.4)	0.92 (0.40–2.09)		
Garre	11 (36.4)(36.4)(27.3)	0.25 (0.07–0.87)		
Other	40 (45.0)(30.0)(17.5)	0.57 (0.28–1.18)		
**SES**		P = 0.9213		
Lowest	139 (56.8)(35.3)(5.8)	1		
Middle	167 (54.5)(35.3)(4.8)	0.77 (0.48–1.23)		
Highest	232 (55.6)(37.1)(4.7)	0.91 (0.59–1.40)		
**Living arrangements**		P<0.001		
Single parent	126 (69.0)(22.2)(3.2)	1		
Both parents	103 (66.0)(23.3)(3.9)	0.96 (0.52–1.75)		
Guardian	23 (52.2)(34.8)(4.3)	0.50 (0.19–1.30)		
Alone or with friend	44 (59.1)(38.6)(0.0)	0.54 (0.26–1.14)		
Spouse	244 (43.4)(47.5)(8.2)	0.26 (0.16–0.43)		
Other	29 (62.1)(34.5)(0.0)	0.63 (0.26–1.53)		
**Belongs to any group?**		P = 0.958		
no	365 (57.0)(35.3)(5.8)	1		
yes	204 (53.4)(36.3)(3.9)	0.99 (0.70–1.41)		
**Peer influence**		P = 0.0284	P = 0.008	
Yes no none	74 (66.2)(23.0)(5.4)	1	1	
Yes to 1 item	128 (53.9)(34.4)(6.3)	0.51 (0.27–0.96)	0.54 (0.28–1.02)	0.059
Yes to 2 or more items	367 (54.2)(38.7)(4.6)	0.46 (0.26–0.82)	0.40 (0.22–0.72)	0.002
**Relationship with parents/guardians**		P<0.001		
Yes no none	347 (48.1)(44.1)(5.8)	1		
Yes to 1 item	40 (65.0)(22.5)(2.5)	2.74 (1.26–5.94)		
Yes to 2 or more items	182 (68.1)(22.5)(4.4)	2.71 (1.78–4.14)		
**Does voluntary work in the community**		P = 0.369		
No	342 (54.4)(37.7)(5.6)	1		
Yes	227 (57.7)(32.6)(4.4)	1.17 (0.83–1.66)		

Model 2: Age- and site-adjusted model for each covariate with *p*<0.10 in Model 1; Model 4: Age, site and socio-demographic adjusted multivariable model including mediating variables with *p*<0.1 after adjusting for Model 3 variables. OR is odds ratio; AOR is adjusted OR.

Table 7 presents results from a linear regression model assessing the factors associated with the “high risk” sexual behaviour composite variable. The estimates from Model 4 show that after adjusting for other socio-demographic and mediating variables, AGYW aged 20–23 years had, on average, higher risky sexual behaviour than their younger counterparts (12–14 years). Marital status was also predictive of high risky sexual behaviour, with the risk being higher among married AGYW. Holding other factors constant, Muslim AGYW had lower risky sexual behaviour than Catholics. In general, AGYW who were in school had, on average, lower risky sexual behaviour than those not in school. Compared to Kikuyu, Luo AGYW exhibited significantly higher, and Garre significantly less, risky sexual behaviour while for other ethnic groups there was no evidence of a difference compared to Kikuyu. Compared to AGYW living with one parent, those living with both parents had lower risky sexual behaviour, while those living alone or with friends, with spouse or with other (unspecified) persons had, on average, higher risky sexual behaviour. Those participating in any social group had lower risky sexual behaviour than those not participating. The results indicated that AGYW whose friends were involved in two or more of the six risk behaviours also had higher risky sexual behaviour. Detailed results from all four step by step models in Tables 4 to 7 are presented in the supplementary material (S3–S6 Tables).

**Table 7:** Factors associated with high risk sexual behaviour composite among AGYW aged 12–23 years.

Variables	High risk sexual behaviour			
	Mean score	Model 2 Estimate (95%CI)	Model 4 Estimate (95%CI)	Pvalue
**Age (years)**		*p*<0.001	*p*<0.001	
12–14	1.0	Ref	Ref	
15–19	2.2	1.17 (0.83–1.50)	0.2 (-0.06–0.47)	0.134
20–23	4.7	3.68 (3.33–4.03)	0.57 (0.21–0.93)	0.002
**Slum area**		*p* = 0.035	*p*<0.001	
Korogocho	2.9	Ref	Ref	
Viwandani	2.8	-0.26 (-0.51–0.02)	-0.42 (-0.64–0.2)	<0.001
**Marital status**		*p*<0.001	*p* = 0.010	
Unmarried	2.1	Ref	Ref	
Currently married	6.3	3.27 (2.95–3.58)	1.21 (0.29–2.13)	0.010
**Religion**		*p*<0.001	*p* = 0.023	
Catholic	3.0	Ref	Ref	
Protestant	2.9	-0.29 (-0.65–0.07)	-0.18 (-0.47–0.10)	0.197
Pentecostal	3.1	0.06 (-0.28–0.4)	0.11 (-0.16–0.37)	0.427
Other Christian	2.9	-0.32 (-0.73–0.1)	-0.14 (-0.46–0.19)	0.413
Muslim	2.0	-1.03 (-1.44–0.62)	-0.69 (-1.22–0.16)	0.011
No Religion	3.5	0.11 (-0.49–0.71)	-0.41 (-0.88–0.06)	0.086
**Schooling**		*p*<0.001	*p*<0.001	
Currently in school	1.4	Ref	Ref	
None/incomplete primary	5.7	3.47 (3.09–3.85)	1.86 (1.48–2.24)	<0.001
Complete primary	5.1	2.87 (2.49–3.25)	1.81 (1.46–2.17)	<0.001
Incomplete secondary	3.7	1.58 (1.0.17–2)	0.95 (0.57–1.32)	<0.001
Complete secondary	4.1	1.68 (1.13–2.23)	0.10 (0.50–1.49)	<0.001
Tertiary	3.5	1.05 (0.39–1.7)	0.85 (0.27–1.43)	0.004
**Ethnicity**		*p*<0.001	*p* = 0.019	
Kikuyu	2.9	Ref	Ref	
Luhya	3.2	0.26 (-0.15–0.66)	0.17 (-0.15–0.49)	0.296
Luo	3.3	0.57 (0.19–0.94)	0.47 (0.18–0.76)	0.002
Kamba	3.1	0.06 (-0.30–0.43)	0.09 (-0.20–0.38)	0.551
Kisii	2.8	-0.27 (-0.85–0.32)	0.03 (-0.44–0.50)	0.899
Garre	1.7	-1.28 (-1.86–0.70)	-0.64 (-1.31–0.04)	0.065
Other	2.1	-0.46 (-0.88–0.04)	-0.1 (-0.56–0.36)	0.659
**Wealth status**		*p* = 0.0852		
Lowest	3.0	Ref		
Middle	3.1	0.06 (-0.29–0.41)		
Highest	2.6	-0.24 (-0.56–0.07)		
**Living arrangements**		*p*<0.001	*p*<0.001	
One parent	2.5	Ref	Ref	
Both parents	1.6	-0.7 (-0.96–0.43)	-0.36 (-0.60–0.11)	0.004
Guardian	2.1	-0.41 (-0.87–0.05)	-0.20 (-0.61–0.22)	0.356
Alone or with friend	4.5	1.41 (0.84–1.98)	0.74 (0.22–1.27)	0.005
Spouse	6.3	3.24 (2.88–3.60)	1.29 (0.34–2.23)	0.008
Other	4.7	1.67 (0.97–2.37)	0.68 (0.04–1.33)	0.037
**Belongs to any group?**		*p*<0.001	*p*<0.001	
No	3.9	Ref	Ref	
Yes	2.0	-1.19 (-1.44–0.95)	-0.59 (-0.79–0.39)	<0.001
**Peer influence**		*p*<0.001	*p*<0.001	
Yes no none	1.9	1	1	
Yes to 1 item	2.1	0.24 (-0.09–0.57)	0.06 (-0.20–0.32)	0.668
Yes to 2 or more items	3.9	1.29 (0.96–1.62)	0.79 (0.52–1.05)	<0.001
**Relationship with parents/guardians**		*p*<0.001		
Yes no none	4.9	Ref		
Yes to 1 item	2.0	-2.18 (-2.6–1.76)		
Yes to 2 or more items	1.9	-2.06 (-2.35–1.77)		
**Does voluntary work in the community**		*p*<0.001		
No	3.2	Ref		
Yes	2.5	-0.47 (-0.71–0.23)		

Model 2: Age- and site-adjusted model for each covariate with *p*<0.10 in Model 1; Model 4: Age, site and socio-demographic adjusted multivariable model including mediating variables with *p*<0.1 after adjusting for Model 3 variables. For “Ref” categories the value is 0.

## Discussion

The persistently high incidence of HIV among adolescent girls and young women in sub-Saharan Africa is galvanizing efforts to respond with prevention programmes. However, in many areas, population level data are missing or lack detail to allow for accurate monitoring of social and behavioural risk factors [21,22]. Using existing data from a detailed study of adolescents, this paper responds to a data gap in terms of understanding sexual risks in the period prior to the implementation of DREAMS interventions among AGYW living in Nairobi’s informal settlements.

Our findings show that sexual behaviours that are known to predispose AGYW to HIV acquisition are prevalent in this population, and increase rapidly from a young age. Among those who had ever had sex (42%), the median age for sexual debut was 16 years, condom use at last sex was 26% and overall about 41% of AGYW were in a relationship where the age difference between them and their sexual partners was at least five years. These findings corroborate earlier findings showing prevalent high risk sexual behaviours among young people living in Nairobi’s slums [7,15,23]. In addition to impacting other social outcomes such as school completion [24] and unintended pregnancies, early sexual debut is associated with a higher risk of HIV [25]. Previous studies suggest that early sexual debut may be driven by early exposure to sexual activity as parents are often forced to share sleeping space with their children, when living conditions are crowded [7]. Space constraints may also force young people to move out of parental homes to their own dwellings prematurely, providing them with opportunities to engage in risk behaviour away from parental supervision [7]. Unexpectedly, we found lower occurrence of multiple sexual partnerships. Earlier studies showed higher levels of multiple sexual partners in the general population [7,26] and we expected this to be the same or similar among AGYW in this population. This observation could be related to under-reporting by AGYW or it could be that multiple sexual partnerships are indeed less prevalent in younger women 15–24 years than in older women (25–49 years) [27,28]. More distally, we found that perceptions of peer involvement in risk behaviour and poor relationships with parents/guardians increased with age. Fewer older adolescents participated in civic or volunteer activities which appeared to be protective against involvement in sexual risk behaviours. These findings are in line with findings by Kabiru and colleagues from the same population that showed that transition to first sex was influenced by place of residence, one’s age, perceived parental monitoring and peer behaviour [15].

We found that a significant proportion of AGYW had been tested for HIV and received their test results. However, a large proportion had never tested and the proportion of AGYW who had a recent test (i.e., in the last 12 months) was quite low [29–31]. An earlier study conducted in 2007, in the same population revealed that only 52% of women aged 15–49 years had ever tested for HIV [30]. In this study we found that slightly more than half (52%) of AGYW had ever tested for HIV and the proportion increased with age. It is generally understood that decision to test might be related to perceived risk such as exposure to unprotected sex [32], and therefore one would expect that the proportions of those who have ever tested would be similar to those who have ever had sex. However, this is not the case especially for AGYW below the age of 20 years. For example, while 29% of adolescents reported that they had ever had sex, a higher proportion (45%) reported that they had ever been tested for HIV. These results need further attention. It could be that sexual experience is under-reported or that mass HIV testing including among sexually inexperienced adolescents might explain the observed difference.

Findings from other studies show that the factors or drivers for engaging in high risk sexual behaviours vary and can be context specific. Through a series of regression analyses we examined potential risk factors for the various known risky sexual behaviours independently but also using a summary measure combining the various variables for risky sexual behaviour as outlined in the methods section of this paper. HIV transmission is reported to be higher in age disparate sexual relations due to weaker power relations and sexual violence [33]. We found that older AGYW (20–23 years) were more likely to have sexual partners who were much older than them compared to those aged 15 to 19 years. This might be related to the desire for material benefits by older AGYW to meet demands for basic needs and other material wants for those with limited financial support from parents and relatives [10]. Viwandani slum has previously been found to have lower burden of HIV [3], better overall educational attainment, better school achievement and more likely for residents to be in gainful employment. We found that AGYW in Viwandani were less likely to be involved in an age-disparate relationship compared to Korogocho slum and those who were in a marital relationship were less likely to be living with a partner who was much older/younger than them.

Generally, results from the composite indicator for high risk sexual behaviour confirm observations from the individual factors discussed above. Older AGYW, those from Korogocho slum, those in marital union, the Luo ethnicity and those who live on their own or with a friend were more likely to be engaged in high risk sexual behaviour. On the other hand, AGYW from the Muslim faith, and those who lived with both parents were significantly less likely to engage in high risk sexual behaviours [34][35].

We found that sexual risk behaviour appears to be a function of age with very low levels of sexual risk behaviour among very young adolescents aged 12–14 years. This finding underscores the need for interventions targeting very young adolescents. Further, like previous studies showing the protective nature of close parental supervision, positive parent-child relationships, and parent-child co-residence [15,23,36,37] suggest the important role that parents can play in HIV prevention programmes targeting AGYW.

The study findings should be interpreted in light of several limitations. First, analysis is based on self-reported information that is subject to biases and recall lapses. For example, as has been found in other studies, there is a possibility of under-reporting of age at sexual debut, age of last sexual partner and number of sexual partners [38,39]. The data are also dated therefore some changes could have occurred since then. However, there being no other data more recently with this population, we believe this is the best and most detailed source for this population, prior to the DREAMS interventions and related impact evaluation studies.

## Conclusions

High risk sexual behaviours among AGYW in Viwandani and Korogocho slums are common and mirror earlier findings on HIV burden that have shown that the HIV prevalence in this population is generally higher than that of non-slum urban and rural areas of the country. Several factors that are strongly related to high risk sexual behaviour may not be amenable to single health interventions, and underpin issues around social support and protection for young people. Peer influence, parental support, neighbourhood influences, and education all point to issues of social protection which are critical in the HIV response for and with AGYW.

## Supporting information

S1 Table Demographic characteristics of the AGYW in Nairobi’s informal settlements of Korogocho. (DOCX)

S2 Table Demographic characteristics of the AGYW in Nairobi’s informal settlements of Viwandani. (DOCX)

S3 Table Factors associated with ever tested for HIV among AGYW aged 12–23 years. (DOCX)

S4 Table Factors associated with condom use at most recent sex among AGYW aged 15–23 years. (DOCX)

S5 Table Factors associated with age disparate sex among AGYW aged 15–23 years. (DOCX)

S6 Table Factors associated with high risk sexual behaviour composite among AGYW aged 12–23 years. (DOCX)

S7 Table Data file with variables used in the analyses. (XLSX)
