# Supplementary material for: Understanding HIV risks among adolescent girls and young women in informal settlements of Nairobi, Kenya: Lessons for DREAMS
Source: PLoS One. 2018 May 31;13(5):e0197479. doi: 10.1371/journal.pone.0197479 (PMC5978990; doi:10.1371/journal.pone.0197479)
Supplement: S1 Table — (DOCX) [file pone.0197479.s001.docx]

**Supporting Information**

**Table S1**. Demographic characteristics of AGYW participants in Nairobi’s informal settlements of Korogocho

|  | **All females** | **12-14 yrs** | **15-19 yrs** | **20-23 yrs** |
| --- | --- | --- | --- | --- |
|  | **N=637** | **N=105** | **N=339** | **N=193** |
| Marital Status |  |  |  |  |
| Unmarried | 551 (86.5) | 105 (100) | 314 (92.6) | 132 (68.4) |
| Currently married | 86 (13.5) | 0 (0.0) | 25 (7.4) | 61 (31.6) |
| Religion |  |  |  |  |
| Catholic | 143 (22.5) | 23 (21.9) | 69 (20.4) | 51 (26.4) |
| Protestant | 126 (19.8) | 17 (16.2) | 72 (21.2) | 37 (19.2) |
| Pentecostal | 99 (15.5) | 15 (14.3) | 58 (17.1) | 26 (13.5) |
| Other Christian | 98 (15.4) | 17 (16.2) | 53 (15.6) | 28 (14.5) |
| Muslim | 133 (20.9) | 30 (28.6) | 68 (20.1) | 35 (18.1) |
| No Religion | 38 (6) | 3 (2.9) | 19 (5.6) | 16 (8.3) |
| Schooling |  |  |  |  |
| Currently school | 377 (59.2) | 105 (100) | 238 (70.2) | 34 (17.6) |
| None/incomplete primary | 75 (11.8) | 0 (0.0) | 25 (7.4) | 50 (25.9) |
| Complete primary | 85 (13.3) | 0 (0.0) | 36 (10.6) | 49 (25.4) |
| Incomplete secondary | 46 (7.2) | 0 (0.0) | 27 (8) | 19 (9.8) |
| Complete secondary | 17 (2.7) | 0 (0.0) | 4 (1.2) | 13 (6.7) |
| Tertiary | 27 (4.2) | 0 (0.0) | 5 (1.5) | 22 (11.4) |
| Missing | 10 (1.6) | 0 (0.0) | 4 (1.2) | 6 (3.1) |
| Ethnicity |  |  |  |  |
| Kikuyu | 255 (40) | 32 (30.5) | 133 (39.2) | 90 (46.6) |
| Luhya | 69 (10.8) | 8 (7.6) | 42 (12.4) | 19 (9.8) |
| Luo | 137 (21.5) | 27 (25.7) | 73 (21.5) | 37 (19.2) |
| Kamba | 37 (5.8) | 7 (6.7) | 16 (4.7) | 14 (7.3) |
| Kisii | 0 (0.0) | 0 (0.0) | 0 (0.0) | 0 (0.0) |
| Garre | 61 (1.0) | 8 (7.6) | 36 (10.6) | 17 (8.8) |
| Other | 78 (2.0) | 23 (21.9) | 39 (11.5) | 16 (8.3) |
| Wealth tertile |  |  |  |  |
| Lowest | 115 (18.1) | 16 (15.2) | 66 (19.5) | 33 (17.1) |
| Middle | 197 (30.9) | 33 (31.4) | 105 (31) | 59 (30.6) |
| Highest | 303 (47.6) | 55 (52.4) | 159 (46.9) | 89 (46.1) |
| Missing | 22 (3.5) | 1 (1) | 9 (2.7) | 12 (6.2) |
| Living arrangements |  |  |  |  |
| Lives with one parent | 165 (25.9) | 25 (23.8) | 97 (28.6) | 43 (22.3) |
| Both parents | 308 (48.4) | 75 (71.4) | 179 (52.8) | 54 (28.0) |
| Guardian | 44 (6.9) | 5 (4.8) | 31 (9.1) | 8 (4.2) |
| Alone or with friend | 21 (3.3) | 0 (0.0) | 6 (1.8) | 15 (7.8) |
| Spouse | 82 (12.9) | 0 (0.0) | 23 (6.8) | 59 (30.6) |
| Other | 17 (2.7) | 0 (0.0) | 3 (0.9) | 14 (7.3) |
