# Supplementary material for: Understanding HIV risks among adolescent girls and young women in informal settlements of Nairobi, Kenya: Lessons for DREAMS
Source: PLoS One. 2018 May 31;13(5):e0197479. doi: 10.1371/journal.pone.0197479 (PMC5978990; doi:10.1371/journal.pone.0197479)
Supplement: S2 Table — (DOCX) [file pone.0197479.s002.docx]

**Supporting Information**

**Table S2**. Demographic characteristics of AGYW participants in Nairobi’s informal settlements of Viwandani

|  | **All females** | **12-14 yrs** | **15-19 yrs** | **20-23 yrs** |
| --- | --- | --- | --- | --- |
|  | **N=753** | **N=162** | **N=311** | **N=280** |
| Marital Status |  |  |  |  |
| Unmarried | 586 (77.8) | 161 (99.4) | 290 (93.3) | 135 (48.2) |
| Currently married | 167 (22.2) | 1 (0.6) | 21 (6.8) | 145 (51.8) |
| Religion |  |  |  |  |
| Catholic | 251 (33.3) | 59 (36.4) | 115 (37) | 77 (27.5) |
| Protestant | 139 (18.5) | 32 (19.8) | 47 (15.1) | 60 (21.4) |
| Pentecostal | 216 (28.7) | 44 (27.2) | 92 (29.6) | 80 (28.6) |
| Other Christian | 71 (9.4) | 16 (9.9) | 21 (6.8) | 34 (12.1) |
| Muslim | 48 (6.4) | 5 (3.1) | 27 (8.7) | 16 (5.7) |
| No Religion | 28 (3.7) | 6 (3.7) | 9 (2.9) | 13 (4.6) |
| Schooling |  |  |  |  |
| Currently school | 417 (55.4) | 159 (98.2) | 222 (71.4) | 36 (12.9) |
| None/incomplete primary | 99 (13.2) | 0 (0.0) | 34 (10.9) | 65 (23.2) |
| Complete primary | 90 (12) | 1 (0.6) | 21 (6.8) | 68 (24.3) |
| Incomplete secondary | 74 (9.8) | 2 (1.2) | 22 (7.1) | 50 (17.9) |
| Complete secondary | 51 (6.8) | 0 (0.0) | 8 (2.6) | 43 (15.4) |
| Tertiary | 17 (2.3) | 0 (0.0) | 2 (0.6) | 15 (5.4) |
| Missing | 5 (0.7) | 0 (0.0) | 2 (0.6) | 3 (1.1) |
| Ethnicity |  |  |  |  |
| Kikuyu | 244 (32.4) | 64 (39.5) | 105 (33.8) | 75 (26.8) |
| Luhya | 89 (11.8) | 13 (8) | 42 (13.5) | 34 (12.1) |
| Luo | 71 (9.4) | 19 (11.7) | 32 (10.3) | 20 (7.1) |
| Kamba | 198 (26.3) | 35 (21.6) | 72 (23.2) | 91 (32.5) |
| Kisii | 71 (9.4) | 16 (9.9) | 22 (7.1) | 33 (11.8) |
| Garre | 8 (1.1) | 1 (0.6) | 4 (1.3) | 3 (1.1) |
| Other | 72 (9.6) | 14 (8.6) | 34 (10.9) | 24 (8.6) |
| Wealth tertile |  |  |  |  |
| Lowest | 176 (23.4) | 29 (17.9) | 77 (24.8) | 70 (25) |
| Middle | 190 (25.2) | 35 (21.6) | 79 (25.4) | 76 (27.1) |
| Highest | 352 (46.8) | 93 (57.4) | 138 (44.4) | 121 (43.2) |
| Missing | 35 (4.7) | 5 (3.1) | 17 (5.5) | 13 (4.6) |
| Living arrangements |  |  |  |  |
| One parent | 179 (23.8) | 36 (22.2) | 102 (32.8) | 41 (14.6) |
| Both parents | 310 (41.2) | 117 (72.2) | 154 (49.5) | 39 (13.9) |
| Guardian | 46 (6.1) | 8 (4.9) | 24 (7.7) | 14 (5) |
| Alone or with friend | 37 (4.9) | 0 (0.0) | 5 (1.6) | 32 (11.4) |
| Spouse | 163 (21.7) | 1 (0.6) | 20 (6.4) | 142 (50.7) |
| Other | 18 (2.4) | 0 (0.0) | 6 (1.9) | 12 (4.3) |
