# Supplementary material for: Understanding HIV risks among adolescent girls and young women in informal settlements of Nairobi, Kenya: Lessons for DREAMS
Source: PLoS One. 2018 May 31;13(5):e0197479. doi: 10.1371/journal.pone.0197479 (PMC5978990; doi:10.1371/journal.pone.0197479)
Supplement: S3 Table — (DOCX) [file pone.0197479.s003.docx]

**Table S3.** Factors associated with ever tested for HIV among AGYW aged 12-23 years

|  | Ever tested for HIV | | | | |
| --- | --- | --- | --- | --- | --- |
|  | Number who ever tested for HIV / N (%) | Model1  unadjusted OR (95%CI) | Model2  AOR (95%CI) | Model3  AOR (95%CI) | Model4  AOR (95%CI) |
| **Age (years)** |  | *p*<0.001 | *p*<0.001 | *p*<0.001 | *p*<0.0001 |
| 12-14 | 39/267 (14.6) | 1 | 1 | 1 | 1 |
| 15-19 | 292/650 (44.9) | 4.78 (3.29-6.94) | 4.74 (3.26-6.89) | 3.22 (2.18-4.75) | 2.89 (1.95-4.29) |
| 20-23 | 387/473 (81.8) | 27.26 (18.01-41.25) | 27.25 (18.01-41.24) | 8.3 (5.07-13.59) | 7.08 (4.28-11.69) |
| **Slum area** |  | *p*=0.869 | *p*=0.530 | *p*=0.121 | *p*=0.523 |
| Korogocho | 331/637 (52.0) | 1 | 1 | 1 | 1 |
| Viwandani | 387/753 (51.4) | 0.98 (0.80-1.21) | 0.92 (0.72-1.18) | 0.81 (0.63-1.06) | 0.92 (0.70-1.20) |
| **Marital Status** |  | *p*<0.001 | *p* <0.001 |  |  |
| Unmarried | 490/1137 (43.1) | 1 | 1 |  |  |
| Currently married | 228/253 (90.1) | 11.97 (7.79-18.39) | 4.83 (3.05-7.65) |  |  |
| **Religion** |  | *p*=0.0042 | *p*=0.005 |  |  |
| Catholic | 203/394 (51.5) | 1 | 1 |  |  |
| Protestant | 141/265 (53.2) | 1.09 (0.80-1.49) | 0.98 (0.68-1.40) |  |  |
| Pentecostal | 171/315 (54.3) | 1.13 (0.84-1.53) | 1.12 (0.80-1.58) |  |  |
| Other Christian | 96/169 (56.8) | 1.24 (0.86-1.78) | 1.14 (0.75-1.75) |  |  |
| Muslim | 69/181 (38.1) | 0.58 (0.4-0.83) | 0.49 (0.32-0.75) |  |  |
| No Religion | 38/66 (57.6) | 1.28 (0.75-2.16) | 0.94 (0.51-1.72) |  |  |
| **Schooling** |  | *p*<0.0001 | *p*<0.001 | *p*<0.001 | *p*<0.001 |
| Currently in school | 252/794 (31.7) |  | 1 | 1 | 1 |
| None/incomplete primary | 140/174 (80.5) | 1 | 3.59 (2.31-5.58) | 1.91 (1.16-3.14) | 1.86 (1.12-3.09) |
| Complete primary | 142/175 (81.1) | 1.2 (0.90-1.6) | 4 (2.54-6.30) | 2.72 (1.69-4.37) | 2.65 (1.63-4.30) |
| Incomplete secondary | 89/120 (74.2) | 1.11 (0.84-1.48) | 2.93 (1.82-4.71) | 2.44 (1.49-3.99) | 2.36 (1.44-3.88) |
| Complete secondary | 52/68 (76.5) | 1.87 (1.28-2.73) | 2.26 (1.19-4.27) | 1.81 (0.94-3.51) | 1.93 (0.99-3.75) |
| Tertiary | 34/44 (77.3) | 3.53 (1.97-6.33) | 2.21 (1.02-4.8) | 2.12 (0.96-4.66) | 2.04 (0.92-4.53) |
| **Ethnicity** |  | *p*=0.0053 | *p*=0.005 |  |  |
| Kikuyu | 271/496 (54.6) | 1 | 1 |  |  |
| Luhya | 85/158 (53.8) | 1.01 (0.70-1.45) | 0.90 (0.6-1.35) |  |  |
| Luo | 110/209 (52.6) | 0.9 (0.65-1.24) | 1.00 (0.69-1.46) |  |  |
| Kamba | 132/239 (55.2) | 1.06 (0.77-1.44) | 0.84 (0.58-1.23) |  |  |
| Kisii | 35/70 (50.0) | 0.85 (0.51-1.39) | 0.69 (0.37-1.29) |  |  |
| Garre | 24/70 (34.3) | 0.43 (0.25-0.73) | 0.33 (0.18-0.60) |  |  |
| Other | 61/148 (41.2) | 0.54 (0.37-0.78) | 0.6 (0.39-0.92) |  |  |
| **Wealth status** |  | *p*=0.0212 | *p*=0.052 |  |  |
| Lowest | 181/311 (58.2) | 1 | 1 |  |  |
| Middle | 194/373 (52.0) | 0.82 (0.6-1.1) | 0.70 (0.50-1.00) |  |  |
| Highest | 309/651 (47.5) | 0.71 (0.54-0.93) | 0.69 (0.5-0.94) |  |  |
| **Living arrangements** |  | *p*<0.0001 | *p*<0.001 | *p*<0.001 | *p*<0.001 |
| One parent | 181/344 (52.6) | 1 | 1 | 1 | 1 |
| Both parents | 202/618 (32.7) | 0.44 (0.33-0.57) | 0.51 (0.38-0.69) | 0.54 (0.4-0.72) | 0.55 (0.41-0.75) |
| Guardian | 42/90 (46.7) | 0.79 (0.49-1.25) | 0.73 (0.44-1.19) | 0.68 (0.41-1.14) | 0.74 (0.44-1.23) |
| Alone or with friend | 42/58 (72.4) | 2.7 (1.42-5.13) | 1.15 (0.58-2.28) | 0.88 (0.44-1.76) | 0.83 (0.41-1.68) |
| Spouse | 221/245 (90.2) | 8.65 (5.36-13.97) | 3.87 (2.31-6.47) | 3.03 (1.70-5.38) | 3.18 (1.78-5.70) |
| Other | 30/35 (85.7) | 5.4 (2.05-14.26) | 2.59 (0.95-7.06) | 1.89 (0.68-5.28) | 1.72 (0.61-4.81) |
| **Belongs to any group?** |  | *p*<0.0001 | *p*=0.038 |  |  |
| No | 377/612 (61.6) | 1 | 1 |  |  |
| Yes | 341/778 (43.8) | 0.48 (0.39-0.60) | 0.77 (0.60-0.99) |  |  |
| **Peer influence** |  | *p*<0.0001 | *p*<0.001 |  | *p*<0.001 |
| Yes no none | 112/292 (38.4) | 1 | 1 |  | 1 |
| Yes to 1 item | 197/485 (40.6) | 1.10 (0.81-1.48) | 1.19 (0.85-1.68) |  | 1.17 (0.82-1.68) |
| Yes to 2 or more items | 409/613 (66.7) | 3.24 (2.42-4.33) | 2.32 (1.66-3.25) |  | 2.01 (1.4-2.88) |
| **Relationship with parents/guardians** | | *p*<0.0001 | *p*<0.001 |  |  |
| Yes no none | 337/445 (75.7) | 1 | 1 |  |  |
| Yes to 1 item | 63/148 (42.6) | 0.23 (0.16-0.34) | 0.41 (0.26-0.63) |  |  |
| Yes to 2 or more items | 318/797 (39.9) | 0.21 (0.16-0.27) | 0.48 (0.35-0.65) |  |  |
| **Does voluntary work in the community** | | *p*=0.906 | *p*=0.181 |  | *p*=0.014 |
| No | 386/747 (51.7) | 1 | 1 |  | 1 |
| Yes | 332/643 (51.6) | 0.99 (0.80-1.22) | 1.18 (0.93-1.51) |  | 1.39 (1.07-1.81) |

Model 1: “Simple” univariable model with each covariate included one at a time; Model 2: Age- and site-adjusted model for each covariate with *p*<0.10 in Model 1; Model 3: Age and site adjusted multivariable model including socio-demographic characteristics with *p*<0.10 in Model 2; Model 4: Age, site and socio-demographic adjusted multivariable model including mediating variables with *p*<0.1 after adjusting for Model 3 variables. OR is odds ratio; AOR is adjusted OR.
