# Supplementary material for: Understanding HIV risks among adolescent girls and young women in informal settlements of Nairobi, Kenya: Lessons for DREAMS
Source: PLoS One. 2018 May 31;13(5):e0197479. doi: 10.1371/journal.pone.0197479 (PMC5978990; doi:10.1371/journal.pone.0197479)
Supplement: S4 Table — (DOCX) [file pone.0197479.s004.docx]

**Table S4.** Factors associated with condom use at most recent sex, among AGYW aged 15-23 years

| Variables | Condom use | | | | |
| --- | --- | --- | --- | --- | --- |
|  | Used condoms / N (%) | Model 1  Unadjusted OR (95%CI) | Model 2  AOR (95%CI) | Model 3  AOR (95%CI) | Model 4*  AOR (95%CI) |
| **Age (years)** |  | *p*=0.0530 | *p*=101 | *p*=0.430 | *p*=0.430 |
| 15-19 | 58/190 (30.5) | 1 | 1 | 1 | 1 |
| 20-23 | 90/379 (23.7) | 0.68 (0.46-1.00) | 0.72 (0.48-1.07) | 1.19 (0.77-1.84) | 1.19 (0.77-1.84) |
| **Slum area** |  | P=0.0550 | P=103 | *p*=0.858 | *p*=0.858 |
| Korogocho | 79/264 (29.9) | 1 | 1 | 1 | 1 |
| Viwandani | 69/305 (22.6) | 0.69 (0.47-1.01) | 0.73 (0.49-1.07) | 1.04 (0.68-1.59) | 1.04 (0.68-1.59) |
| **Marital status** |  | *p*<0.0001 | *p*<0.001 | *p*<0.001 | *p*<0.001 |
| Unmarried | 131/318 (41.2) | 1 | 1 | 1 | 1 |
| Currently married | 17/251 (6.8) | 0.09 (0.05-0.16) | 0.09 (0.05-0.16) | 0.09 (0.05-0.16) | 0.09 (0.05-0.16) |
| **Religion** |  | *p*=0.0640 | *p*=0.0625 |  |  |
| Catholic | 54/172 (31.4) | 1 | 1 |  |  |
| Protestant | 31/107 (29) | 0.83 (0.49-1.42) | 0.84 (0.49-1.44) |  |  |
| Pentecostal | 34/139 (24.5) | 0.67 (0.4-1.11) | 0.7 (0.42-1.18) |  |  |
| Other Christian | 20/73 (27.4) | 0.82 (0.45-1.52) | 0.79 (0.42-1.48) |  |  |
| Muslim | 1/43 (2.3) | 0.05 (0.01-0.38) | 0.05 (0.01-0.36) |  |  |
| No Religion | 8/35 (22.9) | 0.65 (0.27-1.53) | 0.59 (0.25-1.41) |  |  |
| **Schooling** |  | *p*=0.0013 | *p*<0.001 |  |  |
| Currently in school | 48/104 (46.2) | 1 | 1 |  |  |
| None/incomplete primary | 24/155 (15.5) | 0.19 (0.11-0.35) | 0.2 (0.11-0.37) |  |  |
| Complete primary | 38/149 (25.5) | 0.36 (0.21-0.62) | 0.37 (0.21-0.66) |  |  |
| Incomplete secondary | 18/75 (24) | 0.33 (0.17-0.65) | 0.37 (0.18-0.74) |  |  |
| Complete secondary | 12/47 (25.5) | 0.37 (0.17-0.8) | 0.42 (0.19-0.95) |  |  |
| Tertiary | 7/28 (25) | 0.40 (0.15-1.04) | 0.39 (0.15-1.06) |  |  |
| **Ethnicity** |  | *p*=0.5493 | *p*=0.7361 |  |  |
| Kikuyu | 59/211 (28) | 1 | 1 |  |  |
| Luhya | 20/75 (26.7) | 0.98 (0.54-1.78) | 1.01 (0.55-1.85) |  |  |
| Luo | 30/96 (31.3) | 1.13 (0.66-1.91) | 1.07 (0.63-1.82) |  |  |
| Kamba | 26/110 (23.6) | 0.8 (0.47-1.37) | 0.97 (0.55-1.71) |  |  |
| Kisii | 7/27 (25.9) | 0.85 (0.34-2.13) | 1.18 (0.45-3.07) |  |  |
| Garre | 0/11 (0) | 1 (0-0) | 1 (0-0) |  |  |
| Other | 6/39 (15.4) | 0.46 (0.18-1.15) | 0.49 (0.19-1.24) |  |  |
| **Wealth status** |  | *p*=0.5659 | *p*=0.8037 |  |  |
| Lowest | 32/137 (23.4) | 1 | 1 |  |  |
| Middle | 48/165 (29.1) | 1.26 (0.76-2.11) | 1.15 (0.68-1.94) |  |  |
| Highest | 63/238 (26.5) | 1.15 (0.71-1.87) | 1.00 (0.61-1.64) |  |  |
| **Living arrangements** |  | *p*<0.0001 | *p*<0.001 |  |  |
| One parent | 59/126 (46.8) | 1 | 1 |  |  |
| Both parents | 42/103 (40.8) | 0.78 (0.45-1.33) | 0.80 (0.46-1.37) |  |  |
| Guardian | 5/23 (21.7) | 0.34 (0.12-0.99) | 0.34 (0.12-10) |  |  |
| Alone or with friend | 15/44 (34.1) | 0.54 (0.26-1.12) | 0.5 (0.24-1.04) |  |  |
| Spouse | 16/244 (6.6) | 0.07 (0.04-0.13) | 0.07 (0.03-0.12) |  |  |
| Other | 11/29 (37.9) | 0.62 (0.27-1.43) | 0.58 (0.25-1.34) |  |  |
| **Belongs to any group?** |  | *p*=0.3930 | *p*=0.3420 |  |  |
| No | 92/365 (25.2) | 1 | 1 |  |  |
| Yes | 56/204 (27.5) | 1.19 (0.80-1.75) | 1.21 (0.82-1.80) |  |  |
| **Peer influence** |  | *p*=0.3330 | *p*=0.3989 |  |  |
| Yes no none | 17/74 (23) | 1 | 1 |  |  |
| Yes to 1 item | 27/128 (21.1) | 0.84 (0.42-1.68) | 0.77 (0.38-1.56) |  |  |
| Yes to 2 or more items | 104/367 (28.3) | 1.19 (0.66-2.17) | 1.08 (0.59-1.98) |  |  |
| **Relationship with parents/guardians** | | *p*<0.0001 | *p*<0.001 |  |  |
| Yes no none | 56/347 (16.1) | 1 | 1 |  |  |
| Yes to 1 item | 14/40 (35) | 3.23 (1.56-6.69) | 3.22 (1.54-6.76) |  |  |
| Yes to 2 or more items | 78/182 (42.9) | 4.16 (2.75-6.3) | 4.22 (2.70-6.60) |  |  |
| **Does voluntary work in the community** | | *p*=0.1060 | *p*=0.187 |  |  |
| No | 81/342 (23.7) | 1 | 1 |  |  |
| Yes | 67/227 (29.5) | 1.37 (0.94-2.01) | 1.30 (0.88-1.91) |  |  |

Model 1: “Simple” univariable model with each covariate included one at a time; Model 2: Age- and site-adjusted model for each covariate with *p*<0.10 in Model 1; Model 3: Age and site adjusted multivariable model including socio-demographic characteristics with *p*<0.10 in Model 2; Model 4: Age, site and socio-demographic adjusted multivariable model including mediating variables with *p*<0.1 after adjusting for Model 3 variables. OR is odds ratio; AOR is adjusted OR. *No mediating variable progressed to the final model, as such Model 4 is the same as Model 3.
