# Supplementary material for: Understanding HIV risks among adolescent girls and young women in informal settlements of Nairobi, Kenya: Lessons for DREAMS
Source: PLoS One. 2018 May 31;13(5):e0197479. doi: 10.1371/journal.pone.0197479 (PMC5978990; doi:10.1371/journal.pone.0197479)
Supplement: S5 Table — (DOCX) [file pone.0197479.s005.docx]

**Table S5.** Factors associated with age disparate sex among AGYW aged 15-23 years: Results from ordered logistic regression model

| Variables | N (% whose partner's difference was (0-4yr)(5-9yrs)(10+ yrs)) | Age disparity at last sex+ | | | |
| --- | --- | --- | --- | --- | --- |
|  |  | Model1 Unadjusted OR (95%CI) | Model2 AOR (95%CI) | Model3 AOR (95%CI) | Model4 AOR (95%CI) |
| **Age(years)** |  | P=0.680 | P=0.355 | P=0.007 | P=0.005 |
| 15-19 | 190 (53.7)(33.2)(6.8) | 1 | 1 | 1 | 1 |
| 20-23 | 379 (56.7)(36.9)(4.2) | 1.08 (0.75-1.54) | 1.19 (0.83-1.71) | 1.72 (1.16-2.56) | 1.78 (1.19-2.65) |
| **Slum area** |  | P=0.002 | P=0.001 | P=0.045 | P=0.011 |
| Korogocho | 264 (62.9)(28.4)(4.9) | 1 | 1 | 1 | 1 |
| Viwandani | 305 (49.5)(42.0)(5.2) | 0.58 (0.41-0.81) | 0.56 (0.4-0.8) | 0.69 (0.48-0.99) | 0.62 (0.43-0.9) |
| **Marital Status** |  | P<0.0001 | P<0.001 | P<0.001 | P<0.001 |
| Unmarried | 318 (65.7)(26.4)(2.2) | 1 | 1 | 1 | 1 |
| Currently married | 251 (43.0)(47.4)(8.8) | 0.33 (0.23-0.46) | 0.30 (0.21-0.44) | 0.30 (0.21-0.44) | 0.28 (0.19-0.41) |
| **Religion** |  | P=0.2619 | P=0.3453 |  |  |
| Catholic | 172 (60.5)(31.4)(3.5) | 1 | 1 |  |  |
| Protestant | 107 (58.9)(34.6)(4.7) | 0.86 (0.52-1.41) | 0.85 (0.51-1.4) |  |  |
| Pentecostal | 139 (48.9)(46.0)(2.9) | 0.62 (0.39-0.97) | 0.68 (0.43-1.07) |  |  |
| Other Christian | 73 (54.8)(34.2)(5.5) | 0.78 (0.44-1.38) | 0.7 (0.39-1.24) |  |  |
| Muslim | 43 (51.2)(25.6)(18.6) | 0.51 (0.25-1.03) | 0.48 (0.24-0.99) |  |  |
| No Religion | 35 (57.1)(34.3)(5.7) | 0.81 (0.38-1.7) | 0.73 (0.34-1.54) |  |  |
| **Schooling** |  | P=0.0015 | P=0.0025 |  |  |
| Currently in school | 104 (64.4)(24.0)(2.9) | 1 | 1 |  |  |
| None/incomplete primary | 155 (45.2)(45.8)(5.8) | 0.38 (0.22-0.64) | 0.38 (0.22-0.68) |  |  |
| Complete primary | 149 (53.7)(40.9)(4.0) | 0.51 (0.30-0.88) | 0.49 (0.28-0.87) |  |  |
| Incomplete secondary | 75 (53.3)(40.0)(5.3) | 0.49 (0.26-0.93) | 0.56 (0.29-1.08) |  |  |
| Complete secondary | 47 (70.2)(21.3)(8.5) | 0.91 (0.42-1.96) | 1.03 (0.46-2.32) |  |  |
| Tertiary | 28 (67.9)(21.4)(0.0) | 1.37 (0.50-3.77) | 1.15 (0.41-3.27) |  |  |
| **Ethnicity** |  | P=0.1982 | P=0.1794 |  |  |
| Kikuyu | 211 (58.8)(34.6)(3.3) | 1 | 1 |  |  |
| Luhya | 75 (52.0)(36.0)(4.0) | 0.84 (0.49-1.45) | 0.97 (0.56-1.69) |  |  |
| Luo | 96 (57.3)(37.5)(4.2) | 0.89 (0.55-1.45) | 0.83 (0.51-1.36) |  |  |
| Kamba | 109 (57.8)(36.7)(2.8) | 0.97 (0.6-1.55) | 1.22 (0.74-2.02) |  |  |
| Kisii | 27 (51.9)(40.7)(7.4) | 0.68 (0.31-1.49) | 0.92 (0.40-2.09) |  |  |
| Garre | 11 (36.4)(36.4)(27.3) | 0.24 (0.07-0.84) | 0.25 (0.07-0.87) |  |  |
| Other | 40 (45.0)(30.0)(17.5) | 0.5 (0.25-1.02) | 0.57 (0.28-1.18) |  |  |
| **SES** |  | P=0.9100 | P=0.9213 |  |  |
| Lowest | 139 (56.8)(35.3)(5.8) | 1 | 1 |  |  |
| Middle | 167 (54.5)(35.3)(4.8) | 0.86 (0.55-1.36) | 0.77 (0.48-1.23) |  |  |
| Highest | 232 (55.6)(37.1)(4.7) | 0.98 (0.64-1.49) | 0.91 (0.59-1.40) |  |  |
| **Living with who** |  | P<0.0001 | P<0.001 |  |  |
| Single parent | 126 (69.0)(22.2)(3.2) | 1 | 1 |  |  |
| Both parents | 103 (66.0)(23.3)(3.9) | 0.89 (0.49-1.61) | 0.96 (0.52-1.75) |  |  |
| Guardian | 23 (52.2)(34.8)(4.3) | 0.50 (0.19-1.28) | 0.50 (0.19-1.30) |  |  |
| Alone or with friend | 44 (59.1)(38.6)(0.0) | 0.61 (0.30-1.25) | 0.54 (0.26-1.14) |  |  |
| Spouse | 244 (43.4)(47.5)(8.2) | 0.29 (0.18-0.46) | 0.26 (0.16-0.43) |  |  |
| Other | 29 (62.1)(34.5)(0.0) | 0.71 (0.30-1.67) | 0.63 (0.26-1.53) |  |  |
| **Belongs to any group?** | | P=0.952 | P=0.958 |  |  |
| no | 365 (57.0)(35.3)(5.8) | 1 | 1 |  |  |
| yes | 204 (53.4)(36.3)(3.9) | 0.99 (0.70-1.40) | 0.99 (0.70-1.41) |  |  |
| **Peer influence** |  | P=0.1228 | P=0.0284 |  | P=0.008 |
| Yes no none | 74 (66.2)(23.0)(5.4) | 1 | 1 |  | 1 |
| Yes to 1 item | 128 (53.9)(34.4)(6.3) | 0.58 (0.31-1.08) | 0.51 (0.27-0.96) |  | 0.54 (0.28-1.02) |
| Yes to 2 or more items | 367 (54.2)(38.7)(4.6) | 0.57 (0.33-0.98) | 0.46 (0.26-0.82) |  | 0.40 (0.22-0.72) |
| **Relationship with parents/guardians** | | P<0.0001 | P<0.001 |  |  |
| Yes no none | 347 (48.1)(44.1)(5.8) | 1 | 1 |  |  |
| Yes to 1 item | 40 (65.0)(22.5)(2.5) | 2.64 (1.24-5.61) | 2.74 (1.26-5.94) |  |  |
| Yes to 2 or more items | 182 (68.1)(22.5)(4.4) | 2.50 (1.70-3.70) | 2.71 (1.78-4.14) |  |  |
| **Does voluntary work in the community** | | P=0.219 | P=0.369 |  |  |
| No | 342 (54.4)(37.7)(5.6) | 1 | 1 |  |  |
| Yes | 227 (57.7)(32.6)(4.4) | 1.24 (0.88-1.75) | 1.17 (0.83-1.66) |  |  |

Model 1: “Simple” univariable model with each covariate included one at a time; Model 2: Age- and site-adjusted model for each covariate with *p*<0.10 in Model 1; Model 3: Age and site adjusted multivariable model including socio-demographic characteristics with *p*<0.10 in Model 2; Model 4: Age, site and socio-demographic adjusted multivariable model including mediating variables with *p*<0.1 after adjusting for Model 3 variables. OR is odds ratio; AOR is adjusted OR.
