# Supplementary material for: Understanding HIV risks among adolescent girls and young women in informal settlements of Nairobi, Kenya: Lessons for DREAMS
Source: PLoS One. 2018 May 31;13(5):e0197479. doi: 10.1371/journal.pone.0197479 (PMC5978990; doi:10.1371/journal.pone.0197479)
Supplement: S6 Table — (DOCX) [file pone.0197479.s006.docx]

**Table S6.** Factors associated with high risk sexual behaviour composite score variable, among AGYW aged 12-23 years

| Variables |  | High risk sexual behaviour | | | |
| --- | --- | --- | --- | --- | --- |
|  | Mean score | Model 1  Estimate (95%CI) | Model 2  Estimate (95%CI) | Model 3  Estimate (95%CI) | Model 4  Estimate (95%CI) |
| **Age (years)** |  | *p*<0.001 | *p*<0.001 | *p*<0.001 | *p*<0.001 |
| 12-14 | 1.0 | Ref | Ref | Ref | Ref |
| 15-19 | 2.2 | 1.20 (0.87-1.53) | 1.17 (0.83-1.50) | 0.37 (0.10-0.64) | 0.2 (-0.06-0.47) |
| 20-23 | 4.7 | 3.68 (3.34-4.03) | 3.68 (3.33-4.03) | 0.81 (0.45-1.18) | 0.57 (0.21-0.93) |
| **Slum area** |  | *p*=0.305 | *p*=0.035 | *p*<0.001 | *p*<0.001 |
| Korogocho | 2.9 | Ref | Ref | Ref | Ref |
| Viwandani | 2.8 | -0.15 (-0.44-0.14) | -0.26 (-0.51--0.02) | -0.65 (-0.87--0.43) | -0.42 (-0.64--0.2) |
| **Marital status** |  | *p*<0.001 | *p*<0.001 | *p*=0.003 | *p*=0.010 |
| Unmarried | 2.1 | Ref | Ref | Ref | Ref |
| Currently married | 6.3 | 4.19 (3.89-4.48) | 3.27 (2.95-3.58) | 1.44 (0.49-2.38) | 1.21 (0.29-2.13) |
| **Religion** |  | *p*=0.0002 | *p*<0.001 | *p*=0.0103 | *p*=0.023 |
| Catholic | 3.0 | Ref | Ref | Ref | Ref |
| Protestant | 2.9 | -0.11 (-0.53-0.31) | -0.29 (-0.65-0.07) | -0.20 (-0.49-0.09) | -0.18 (-0.47-0.10) |
| Pentecostal | 3.1 | 0.09 (-0.3-0.49) | 0.06 (-0.28-0.4) | 0.11 (-0.16-0.38) | 0.11 (-0.16-0.37) |
| Other Christian | 2.9 | -0.10 (-0.59-0.38) | -0.32 (-0.73-0.1) | -0.18 (-0.51-0.16) | -0.14 (-0.46-0.19) |
| Muslim | 2.0 | -0.96 (-1.43--0.48) | -1.03 (-1.44--0.62) | -0.83 (-1.38--0.28) | -0.69 (-1.22--0.16) |
| No Religion | 3.5 | 0.57 (-0.13-1.27) | 0.11 (-0.49-0.71) | -0.36 (-0.84-0.13) | -0.41 (-0.88-0.06) |
| **Schooling** |  | *p*<0.001 | *p*<0.001 | *p*<0.001 | *p*<0.001 |
| Currently in school | 1.4 | Ref | Ref | Ref | Ref |
| None/incomplete primary | 5.7 | 4.27 (3.93-4.61) | 3.47 (3.09-3.85) | 2.2 (1.82-2.58) | 1.86 (1.48-2.24) |
| Complete primary | 5.1 | 3.69 (3.35-4.03) | 2.87 (2.49-3.25) | 2.05 (1.69-2.41) | 1.81 (1.46-2.17) |
| Incomplete secondary | 3.7 | 2.26 (1.86-2.66) | 1.58 (1.0.17-2) | 1.11 (0.72-1.5) | 0.95 (0.57-1.32) |
| Complete secondary | 4.1 | 2.62 (2.11-3.14) | 1.68 (1.13-2.23) | 1.24 (0.74-1.74) | 0.10 (0.50-1.49) |
| Tertiary | 3.5 | 2.11 (1.48-2.74) | 1.05 (0.39-1.7) | 1.05 (0.45-1.64) | 0.85 (0.27-1.43) |
| **Ethnicity** |  | *p*<0.0001 | *p*<0.001 | *p*=0.0479 | *p*=0.019 |
| Kikuyu | 2.9 | Ref | Ref | Ref | Ref |
| Luhya | 3.2 | 0.28 (-0.2-0.76) | 0.26 (-0.15-0.66) | 0.13 (-0.20-0.46) | 0.17 (-0.15-0.49) |
| Luo | 3.3 | 0.43 (-0.01-0.86) | 0.57 (0.19-0.94) | 0.41 (0.11-0.71) | 0.47 (0.18-0.76) |
| Kamba | 3.1 | 0.23 (-0.18-0.65) | 0.06 (-0.30-0.43) | 0.09 (-0.21-0.38) | 0.09 (-0.20-0.38) |
| Kisii | 2.8 | -0.17 (-0.83-0.50) | -0.27 (-0.85-0.32) | -0.01 (-0.49-0.47) | 0.03 (-0.44-0.5) |
| Garre | 1.7 | -1.21 (-1.88--0.54) | -1.28 (-1.86--0.70) | -0.69 (-1.38-0.01) | -0.64 (-1.31-0.04) |
| Other | 2.1 | -0.68 (-1.17--0.19) | -0.46 (-0.88--0.04) | -0.08 (-0.55-0.40) | -0.1 (-0.56-0.36) |
| **Wealth status** |  | *p*=0.0014 | *p*=0.0852 |  |  |
| Lowest | 3.0 | Ref | Ref |  |  |
| Middle | 3.1 | -0.01 (-0.42-0.40) | 0.06 (-0.29-0.41) |  |  |
| Highest | 2.6 | -0.4 (-0.77--0.04) | -0.24 (-0.56-0.07) |  |  |
| **Living arrangements** |  | *p*<0.0001 | *p*<0.001 | *p*<0.001 | *p*<0.001 |
| One parent | 2.5 | Ref | Ref | Ref | Ref |
| Both parents | 1.6 | -0.88 (-1.15--0.61) | -0.7 (-0.96--0.43) | -0.41 (-0.66--0.16) | -0.36 (-0.60-0.11) |
| Guardian | 2.1 | -0.38 (-0.86-0.10) | -0.41 (-0.87-0.05) | -0.27 (-0.7-0.15) | -0.20 (-0.61-0.22) |
| Alone or with friend | 4.5 | 2.03 (1.45-2.60) | 1.41 (0.84-1.98) | 0.84 (0.31-1.38) | 0.74 (0.22-1.27) |
| Spouse | 6.3 | 3.85 (3.51-4.19) | 3.24 (2.88-3.60) | 1.09 (0.12-2.06) | 1.29 (0.34-2.23) |
| Other | 4.7 | 2.29 (1.57-3.00) | 1.67 (0.97-2.37) | 0.84 (0.18-1.50) | 0.68 (0.04-1.33) |
| **Belongs to any group?** |  | *p*<0.001 | *p*<0.001 |  | *p*<0.001 |
| No | 3.9 | Ref | Ref |  | Ref |
| Yes | 2.0 | -1.87 (-2.14--1.60) | -1.19 (-1.44--0.95) |  | -0.59 (-0.79--0.39) |
| **Peer influence** |  | *p*<0.0001 | *p*<0.001 |  | *p*<0.001 |
| Yes no none | 1.9 | 1 | 1 |  | 1 |
| Yes to 1 item | 2.1 | 0.18 (-0.19-0.55) | 0.24 (-0.09-0.57) |  | 0.06 (-0.20-0.32) |
| Yes to 2 or more items | 3.9 | 1.94 (1.59-2.3) | 1.29 (0.96-1.62) |  | 0.79 (0.52-1.05) |
| **Relationship with parents/guardians** | |  | *p*<0.001 |  |  |
| Yes no none | 4.9 | Ref | Ref |  |  |
| Yes to 1 item | 2.0 | -2.91 (-3.34--2.48) | -2.18 (-2.6--1.76) |  |  |
| Yes to 2 or more items | 1.9 | -3.06 (-3.33--2.79) | -2.06 (-2.35--1.77) |  |  |
| **Does voluntary work in the community** | |  | *p*<0.001 |  |  |
| No | 3.2 | Ref | Ref |  |  |
| Yes | 2.5 | -0.68 (-0.97--0.4) | -0.47 (-0.71--0.23) |  |  |

Model 1: “Simple” univariable model with each covariate included one at a time; Model 2: Age- and site-adjusted model for each covariate with *p*<0.10 in Model 1; Model 3: Age and site adjusted multivariable model including socio-demographic characteristics with *p*<0.10 in Model 2; Model 4: Age, site and socio-demographic adjusted multivariable model including mediating variables with *p*<0.1 after adjusting for Model 3 variables. For “Ref” categories the value is 0.
